# Justifiability and Animal Research in Health: Can Democratisation Help Resolve Difficulties?

**DOI:** 10.3390/ani8020028

**Published:** 2018-02-14

**Authors:** Shaun Yon-Seng Khoo

**Affiliations:** Center for Studies in Behavioral Neurobiology/Groupe de Recherche en Neurobiologie Comportementale, Department of Psychology, Concordia University, Montreal, QC H4B 1R6, Canada; shaun.khoo@concordia.ca; Tel.: +1-514-848-2424 (ext. 5736)

**Keywords:** animal ethics, consequentialism, harm-benefit analysis, justification, democratisation, ethical consumerism, animal ethics committees

## Abstract

**Simple Summary:**

Scientists justify animal use in medical research because the benefits to human health outweigh the costs or harms to animals. However, whether it is justifiable is controversial for many people. Even public interests are divided because an increasing proportion of people do not support animal research, while demand for healthcare that is based on animal research is also rising. The wider public should be given more influence in these difficult decisions. This could be through requiring explicit disclosure about the role of animals in drug labelling to inform the public out of respect for people with strong objections. It could also be done through periodic public consultations that use public opinion and expert advice to decide which diseases justify the use of animals in medical research. More public input will help ensure that animal research projects meet public expectations and may help to promote changes to facilitate medical advances that need fewer animals.

**Abstract:**

Current animal research ethics frameworks emphasise consequentialist ethics through cost-benefit or harm-benefit analysis. However, these ethical frameworks along with institutional animal ethics approval processes cannot satisfactorily decide when a given potential benefit is outweighed by costs to animals. The consequentialist calculus should, theoretically, provide for situations where research into a disease or disorder is no longer ethical, but this is difficult to determine objectively. Public support for animal research is also falling as demand for healthcare is rising. Democratisation of animal research could help resolve these tensions through facilitating ethical health consumerism or giving the public greater input into deciding the diseases and disorders where animal research is justified. Labelling drugs to disclose animal use and providing a plain-language summary of the role of animals may help promote public understanding and would respect the ethical beliefs of objectors to animal research. National animal ethics committees could weigh the competing ethical, scientific, and public interests to provide a transparent mandate for animal research to occur when it is justifiable and acceptable. Democratic processes can impose ethical limits and provide mandates for acceptable research while facilitating a regulatory and scientific transition towards medical advances that require fewer animals.

## 1. Introduction

Animal research is frequently considered justifiable based on a consequentialist calculus that invokes cost-benefit or harm-benefit analysis [1]. These ethical frameworks are formalised throughout the developed world with explicit statements in regulations and guidelines requiring researchers to justify their use of animals based on benefits to humans, animals, or the environment. These frameworks rely on researchers presenting evidence that their research may lead to benefits, such as addressing an unmet medical need, but many members of the public disagree. Opinion polls in the US and the UK show that the proportion of adults who believe medical research involving animals is morally acceptable has been falling since 2002 [2]. However, this trend in public opinion is at odds with the rising demand for healthcare [3], including drugs that are tested on animals as part of regulatory requirements [4]. Despite the trend in public opinion, current ethical and regulatory frameworks lack the capacity to reduce the scope and volume of animal research because the consequentialist calculus is too rough and imprecise. Options for democratising animal research ethics should be considered, including drug labelling to educate the public and public consultation by national animal ethics committees to engage the public in deciding when animal research is justified.

## 2. Ethical Flexibility

The research community has largely adopted the consequentialist ethics that were used to shift attitudes against animal research. In the 1970s, Peter Singer famously argued that the suffering of animals should not be given less weight than the suffering of humans, a view that he believed should end the vast majority of animal research [5,6]. However, others have argued that animal research is necessary to maximise goods and avoid harms [7]. Indeed, the benefits of animal research are the primary argument advanced by scientists who use animals [8], even though moral philosophers as varied as deontologists, ecofeminists, and virtue ethicists may find it unconvincing [9,10,11,12]. The consequentialist framework has now broadly been adopted by the research community, with animals given ethical standing and being included in cost-benefit rubrics. Animal ethics processes require justification for research projects (benefit) and address costs by embedding the 3Rs of replacement, reduction, and refinement into regulations and guidelines [13]. However, animal research continues, leaving its opponents dissatisfied.

One issue is that the calculation of ethical costs to animals and the benefits of animal research are so rough and imprecise (if they are even possible) [6,14] that they can lead to almost any conclusion. An optimist could argue that since a particular improvement in healthcare could benefit humans in perpetuity, but takes only a finite number of animals to achieve, almost any project could be justifiable. Moreover, the practical benefits of a research project, like the results of individual experiments, are hard to predict and may be more useful than anticipated. For example, in 2016, there were eight first-in-class drugs approved that perhaps may not have been possible without animal use (see Table 1) [15]. Opponents of animal research place less weight on the benefits and greater weight on the costs [11], or criticise researchers for having the opposite bias [14]. The flexibility and imprecision of the consequentialist calculus makes it very difficult to reject projects on ethical grounds, provided they have a properly articulated rationale.

In theory, there should be a point where the benefit side of the consequentialist calculus is outweighed by the costs to the animals. If it were possible to quantify a benefit, at least in health, it should probably be based on the World Health Organization’s measure of disease burden, disability-adjusted life-years (DALYs). Moral philosophers, however, have argued that certain conditions should not be targeted based on the quality of the disorder or disease being researched, rather than the quantity of the benefit. For example, the ethics of better therapies for insomnia or experiments in psychology have been questioned [11,14], despite the fact that psychological disorders account for about a quarter of Europe’s DALYs [16]. In fact, in 2015, depression was the 10th biggest contributor to disease burden, with 12 million DALYs lost [17]. Even if it is assumed that all of the 50–100 million vertebrates used in experiments per year [18,19] are used for the development of 30 new drugs [15], the 169 million DALYs lost to mental and substance use disorders [17] eclipses the 2–3 million animals each new drug requires on average. Although the consequentialist calculus is supposedly a quantitative exercise [14], no regulatory guidelines or ethics committees have been reported to utilise a cut-off based on DALYs or any other objective measure for approving projects.

### Alternative Ethical Frameworks

There are alternative ethical frameworks to consequentialism, but these have not been formally adopted and leave the question of justifiability contested. Deontological or rights-based frameworks and virtue ethics frameworks may also still consider consequences. In the extreme, a rights-based framework calls for the total abolition of all animal research, as Tom Regan argued based on the idea that animals have inherent value as living creatures [42]. However, a minimalist view of animal rights would be that animals should have the right to freedom from “useless pain or misery” [43]. The usefulness of the animal’s suffering here invokes consequentialism—it is the benefit for science and health that permits the research. Rights-based frameworks have also struggled to gain formal acceptance as courts have struggled with the idea of granting rights to animals [44], so though the framework may have philosophical value, few animal ethics systems formally adopt the principle. Similarly, virtue ethicists may argue that animal research is cruel and therefore immoral, using reasoning that is similar to consequentialist approaches [10]. However, it can also be argued that animal research is compassionate when conducted for the purposes of improving healthcare, perhaps based on discounting animal interests because of partiality to other human ties [9,45], but this potentially leaves virtue ethics in a similar impasse to consequentialism as individuals must weigh their compassion for patients against compassion for animals. There is as yet no ethical framework that can clearly and uncontroversially delineate justifiable and unjustifiable research in a way that is significantly more satisfactory from a philosophical and practical perspective.

## 3. Consequentialism and Public Interest

The conclusions of different consequentialist philosophers, scientists and perceptions of the public are often in disagreement. Scientists often argue that a study is justifiable because there is unmet medical need, but it is unclear whether members of the public (or consequentialist philosophers) would agree that every unmet medical need warrants the use of animals in medical research. In general, a majority of people believe animal research is ethically acceptable [2,46], but this includes no fine-grained information about particular conditions or approaches. A purely consequentialist ethical approach would probably target research funding in accordance with burden of each disease or disorder, but that is not the case in reality. Instead, funding for research into different disorders is skewed by societal attitudes, with certain medical conditions attracting an excess of funding while others are underfunded relative to their disease burden [47,48,49]. Societal attitudes are also less supportive of certain techniques, like genetic modification, in animal research [50].

The public might consider other factors when deciding whether animal research is acceptable for a particular condition. For example, it may be seen as more justifiable to use animals to improve treatments for conditions that affect earlier stages of life rather than later stages because there is potentially a greater benefit. Disorders that are perceived to have an element of choice, like addiction [51], could also conceivably be seen as less ethical choices for animal use in medical research. However, people with addiction still seek treatment, including medication, and this demand for new, more effective treatments has previously been argued as a justification for further research [52].

The public’s demand for healthcare continues to rise suggesting a strong public interest in maintaining and improving current standards. Healthcare spending is rising at a rate above inflation, with large increases in pharmaceutical spending [3,53]. Interviews with stakeholders about pharmaceuticals policy show that they are also concerned about equitable access, for instance patients with rare disorders [54]. Rare (or “orphan”) disorders pose a difficulty for consequentialist ethics because benefits are accrued by a smaller number of people, thus shifting the consequentialist calculus against lines of research. These rare diseases may have no effective therapies available and thus attract significant sympathy and political advocacy, as was the case for the controversial approval of eteplirsen [55]. Therefore, it appears that the public places at least some weight on the more abstract benefit of equitable access to healthcare that may impact or override an otherwise unfavourable consequentialist calculus.

Evaluations of animal research ethics must therefore weigh a multitude of competing viewpoints. Surveys have shown that public opinion is turning against animal research, with support dropping from 75% to 66% in the UK and from 63% to 51% in the US between 2002 and 2016 [2,46]. One Canadian survey also found minority (44%) public support for animal research that benefitted humans but harmed animals. Respondents were then presented with simplified typical arguments against animal research and virtually all undecided individuals were convinced animal research should not be supported [56]. On the other hand, the behaviour of the public in seeking healthcare and demanding new therapies, sometimes before they have been clinically tested [57], suggests that (insofar as animal research is currently necessary for drug development) there is an indirect public interest in continuing to do animal research. Although some philosophers argue that all medical research could be done morally in humans or using alternatives, scientists have consistently argued that animals are currently necessary to make medical advances [8,58]. If it continues, animal activists will continue to campaign against their work, potentially using aggressive protest techniques like targeting individual scientists [46,59]. Democratisation can help society navigate the uncertainty of the consequentialist calculus and balance it with other public interest considerations through individual action and enhanced opportunities for deliberative participation [60].

## 4. Ethical Health Consumerism

One form of democratisation is through ethical consumerism. Moral philosophers have previously argued for changes in individual behaviour to improve the treatment of animals. For example, consequentialists and virtue ethicists have made arguments to support vegetarianism because withdrawing support for the meat industry can either help reduce animal suffering or is otherwise compassionate and generous [10,61,62]. There is also historical precedent for similar actions in healthcare. For example, in early 19th century Britain, doctors who performed vivisection were subject to boycotts out of fear that they lacked sensitivity and compassion [63].

In the late 20th century, it was suggested (perhaps sarcastically) that patients concerned with animal research should give their doctor an advanced directive refusing treatments derived from animal research, i.e., all of them [64]. More recently, it was proposed to label medicines in the UK as tested on animals to inform the public of the role of preclinical animal work [65]. However, animal activists opposed the labelling initiative, citing concerns that patients may not comply with medication because it had been tested on animals and arguing that animal research made no meaningful contribution to drug development anyway [66]. In the same vein, animal activists have also argued (as a reductio ad absurdum) that supporters of animal research should volunteer to be experimented upon should they lose mental capacity [67].

However, the application of ethical consumerism to healthcare has the capacity to inform the public and to democratise the consideration of the consequentialist calculus. Non-compliance with medication may be a concern for clinicians, but refusing treatment is well within a patient’s rights [68]. There is currently no external labelling indicating that a given drug was tested on animals and no further information in the product information sheet. Moreover, current labels do not even provide adequate information for the 5–10% of patients who are vegetarian or vegan about the suitability of a drug’s ingredients [69]. Given the state of public opinion, opening healthcare to ethical consumerism through labelling and disclosure can instead be seen as a means of respecting the ethical views of a significant minority of the population. Currently, adult patients with decision-making capacity can refuse treatments that are inconsistent with their religious, ethical, or other personal beliefs [70,71]. Non-disclosure regarding the role of animals in a treatment’s development implicitly denies the validity of patients’ ethical beliefs and their right to give informed consent or refusal.

### Objections to Ethical Health Consumerism

Animal activists have argued that a simple external label may not be sufficient on its own [66], but the proposal could be refined by requiring pharmaceutical companies to provide a plain-language summary of the role animals have played in a drug’s development. Surveys have also been cited suggesting that pharmaceutical companies only do animal research to satisfy regulators [72]. On the other hand, industry studies suggest that results from animals are imprecise, but still useful predictors of the likelihood of adverse outcomes in clinical trials [73,74]. Since the reliability and usefulness of animal results differs based on circumstances (e.g., species and organ) [73], the pharmaceutical company that developed the drug is best-placed to summarise the role of animal research for an individual drug. However, care must be taken by regulators when reviewing these statements because there have been cases of researchers selectively reporting data from animal research that results in ineffective or unsafe compounds proceeding to clinical trials [75,76] just as regulators must take care to ensure clinical trials are well designed [77]. Requiring labelling and a plain-language summary of the role of animals in a drug’s development could help the public understand the contribution that animals have made and to then evaluate whether the benefits to them have outweighed to costs to the animals.

Research on consumer behaviour suggests clinician and animal activist concerns about medical non-compliance or refusal are not likely to be a major issue. Consumer behaviour is affected by many factors other than ethical labels [78,79]. The expression of ethical concerns does not necessarily translate into changes in behaviour and consumers who do change their behaviour represent a minority [78]. For example, one study found an ‘organic’ label had no significant effect on choice of chocolate [80]. However, this has not prevented several ethical consumer movements targeting animal welfare and environmental issues, such as egg-laying hens, from achieving some success [81,82,83].

Moral philosophers have also questioned the possibility of refraining from being a party to animal research. It has been argued that animal research is too deeply entrenched in corporate and academic research centres for individuals to withdraw their support with any hope of affecting change [10]. However, a small minority of patients who demand an alternative type of care can help drive change, such as with the development of bloodless medicine for Jehovah’s Witnesses [84]. Moderates concerned with animal use could approach the issue by accepting life-saving treatments, but refusing non-critical treatments and ensuring they implement an advance care directive to limit the amount of medical care they receive if they lose decision-making capacity. Even a small number of animal research objectors could be enough to encourage the regulatory and scientific developments needed for animal-free medical advances.

## 5. Participatory Decision-Making: Are National Animal Ethics Committees Needed?

Another process for democratising the consequentialist calculus of animal research is to give the public direct input into deliberative processes [60]. Researchers have variously argued for enhanced approaches to project assessment [18] and for political processes as necessary for the determination of what research is acceptable [85]. Electoral and parliamentary processes can initiate change, but legislation banning animal research is too blunt and inflexible to incorporate society’s diverse views on animal research and the varying circumstances for different conditions. Fine-grained information about what kinds of research are acceptable is necessary to ensure that animal research meets public expectations.

Current institutional ethics review processes have been criticised because they do not adequately ensure that animal research is valid or ethical. For example, they may not adequately assess the scientific validity of a project before giving approval [86] and many animal welfare officers at Australian and Dutch universities feel that 3Rs opportunities remain unused [87,88]. There are also anecdotal reports that rejection of projects at the ethics committee stage never or almost never happens [89]. This suggests that the animal research approval processes used at the institutional level for individual projects are incapable of determining when animal costs outweigh potential benefits.

A national animal ethics committee, organised by funders and/or regulators, could democratically engage the public and weigh scientific evidence to determine what kinds of animal research are acceptable. Historically, these kinds of ‘boundary organisations’ have sat at the intersection of science and politics and help to resolve competing or opposing interests while simultaneously pursuing both [90,91]. The challenge is in designing a deliberative space or process that can adequately communicate science and give the public an appropriate degree of influence on decision-making [92]. Although there is already some democratisation of animal ethics procedures with independent or unaffiliated members being included in institutional animal ethics committees in several jurisdictions, the effectiveness of these roles has been criticised for lacking in independence or representativeness [89]. There are also challenges in terms of education and training and the inherent difficulty of representing the views of a public that is divided on animal research [93]. A national animal ethics committee could overcome some of these difficulties through standard public consultation processes (e.g., surveys, hearings, written submissions) and allow institutional animal ethics committees to focus on issues with individual projects rather than resolving macro-level ethical and public interest issues. Public engagement with these processes could be built through programs like the Concordat on Openness on Animal Research in the UK, which encourages signatories to better communicate their use of animals (including its limitations) through annual reporting and making online statements about their animal use and policies [94]. Public participants in surveys, focus groups, or hearings, may then be more engaged and educated about the issue.

The ongoing deadlock between animal activists and researchers suggests that animal research is a good candidate for participatory decision-making [95]. If public opinion follows current trends [2], then the deadlock and hostility towards scientists will only worsen over time. It may be costly because every citizen is potentially a stakeholder due to an interest in either healthcare or animal welfare, but online participation can reduce costs. Conducting surveys of representative samples can also help to reduce costs while providing accurate information on public opinion and reducing vulnerability to political campaigns. Animal research ethics may also require participants to understand highly technical concepts, but the issue could also attract many passionate patient advocates and animal activists which could ameliorate these difficulties through public education [95]. Moreover, both sides stand to benefit. Scientists would have an explicit democratic mandate to conduct particular lines of research. Objectors to animal research would, subject to current trends in public opinion, be able to gradually decrease the use of animals in research and lobby for changes like repealing requirements to use animals in drug development or increasing funding for 3Rs developments.

## 6. Accelerating Ethical Progress

The democratisation of decisions about animal research is a logical consequence of the general democratisation of science. As science becomes more open (e.g., through open access and open data practices) and participatory (e.g., citizen science), it is reasonable that information and decision-making processes about basic research should also be more available. Democratisation can improve animal research ethics because it will provide a process for clearly delineating justifiable and unjustifiable uses of animals and is likely to accelerate 3Rs implementation. Similarly, drug labelling with plain language summaries of the usefulness (and limitations) of preclinical animal research can help to educate the public about the role of animal research in their lives. This enables individuals to make decisions consistent with their ethical beliefs about their healthcare and refusals on ethical grounds can help accelerate improvements in animal welfare principles such as the 3Rs. If data on the rate at which patients refuse treatments on ethical grounds is collected, it can discourage researchers and biomedical companies from developing treatments that the public finds ethically unacceptable. It can also raise awareness in patients, who can use their powerful lobbying and advocacy groups to push for political changes such as repealing the regulatory requirement to test drugs on animals, as they have done with right-to-try laws [57].

Direct consultation and participatory decision-making through national animal ethics committees would likely be framed (at least initially) in consequentialist terms—cost or harm-benefit—and would require transparent weightings for different interests or viewpoints (e.g., biomedical scientists, patients, public opinion). Over time, it would be expected that the number of lines of animal research that would be considered acceptable would decrease. This would occur as long as the public is given genuine influence over decisions because current trends in public opinion show increasing opposition to animal research [2,46]. Scientists would also need explicit protections from sudden changes in sentiment, such as grandfathering of ethical approvals for already funded or reviewed projects and limits to the rate of change that could suddenly impact the biomedical workforce. However, the effect of democratisation and current trends would be to benefit animals who would be used in fewer experiments, patients who want to receive treatments consistent with their ethical beliefs, and scientists who will have some certainty that if they are working on animal projects that they have a democratic mandate to do so.

Political engagement with animal research is already promoting improved animal welfare. For example, political engagement and regulatory developments in Europe are already pushing scientists to develop and improve animal welfare standards [96]. Political and regulatory pressure must also be accompanied by the necessary resources to study and improve animal welfare, which may be very scarce in a regulatory and funding system focused primarily on human health needs. While practical scientific impediments to eliminating animal research completely may continue for some time, such as the impossibility of understanding behavioural processes without animals [8], increasing the level of information available to the public and their ability to participate in decision-making about the animal research that their governments support is likely to accelerate implementation of 3Rs principles.

## 7. Conclusions

The current consequentialist ethical framework and institutional approval processes for animal research cannot satisfactorily resolve the question of when animal costs outweigh potential human benefits. The consequentialist calculus is too rough and imprecise to produce clear, reproducible conclusions and the result is that there is a significant divide within and between public opinion on animal research, public demand for healthcare, and scientific opinion. Methods of democratisation can help stakeholders with diverse and conflicting viewpoints give input into what kinds of benefits may justify the use of animals in research. Facilitating ethical health consumerism through labelling disclosure of animal use in the development of drugs or convening a national animal ethics committee to determine which purposes are acceptable can provide the fine-grained data required to guide animal researchers to the most ethical projects. Democratising deliberations on the justifiability of animal research can help ensure that the interests of animal researchers and animal activists are balanced in accordance with public expectations and can potentially facilitate changes that would enable medical advances that use fewer animals.

## Figures and Tables

**Table 1:** Basic and preclinical animal research and first-in-class drugs approved by the United States Food and Drug Administration in 2016.

Trade Name (Drug)	Disease	Animal Use
Defitelio (defibrotide sodium)	Hepatic veno-occlusive disease afterhaematopoietic stem cell transplantation *	Derived from the intestinal mucosa of pigs, defibrotide has been tested in several cell lines and in animals, such as mice [20,21,22].
Exondys 51 (eteplirsen)	Duchenne muscular dystrophy *	Eteplirsen works by causing exon skipping to correct a genetic mutation. Animal studies on the approach used mice and dogs [23]. However, its approval using a surrogate marker of efficacy in humans is controversial [24].
Ocaliva (obeticholic acid)	Primary biliary cirrhosiss *	Obeticholic acid is a farnesoid-X receptor agonist. The farnesoid-X receptor was recommended as a drug target based on studies in rats [25,26,27].
Spinraza (nusinersen)	Spinal muscular atrophy *	Nusinersen (like eteplirsen) works by modulating gene splicing to increase levels of a protein affected by an inherited genetic mutation. It was advanced to clinical trials based on work in mice and non-human primates [28,29,30].
Venclexta (venetoclax)	Chronic lymphocytic leukemia *	Targets the Bcl2 receptor, based on basic research into apoptosis and mouse cancer models [31,32,33].
Xiidra (lifitegrast)	Dry eye disease	Dry eye disease can affect animals like dogs, cats, and horses and animal models have provided evidence of an inflammatory role. Lifitegrast reduces inflammation by preventing LFA-1/ICAM-1 interactions and has been tested indogs and mice [34,35,36].
Zinbryta (daclizumab)	Multiple sclerosis	Daclizumab was originally developed as immune suppressant for transplant patients and based on studies in mice showing mechanisms to suppress autoimmune responses. Human clinical studies facilitated its translation for multiple sclerosis [37,38].
Zinplava (bezlotoxumab)	Clostridium difficile infection	Bezlotoxumab neutralises C. *difficile* toxin B. Early work involved characterising the effect of toxin B in animals like hamsters and rabbits and using rabbits to generate antibodies against toxin B [39,40,41].

* “Orphan” or rare diseases affecting fewer than 200,000 Americans often have limited treatment options or no drug treatment available.
